# Pain in the Prehospital Setting in Rwanda: Results of a Mixed-Methods Quality Improvement Project

**DOI:** 10.1155/2020/3284623

**Published:** 2020-09-18

**Authors:** A. Rosenberg, E. Uwitonze, M. Dworkin, J. P. D. Guidry, T. Cyuzuzo, D. Banerjee, K. McIntyre, K. Carlyle, J. M. Uwitonze, I. Kabagema, T. Dushime, S. Jayaraman

**Affiliations:** ^1^Department of Surgery, Virginia Commonwealth University, School of Medicine, Richmond, VA, USA; ^2^Service d'Aide Medicale d'Urgence, Rwanda Ministry of Health, Kigali, Rwanda; ^3^Thomas Jefferson University Sidney Kimmel School of Medicine, Philadelphia, PA, USA; ^4^Richard T. Robertson School of Media and Culture, Virginia Commonwealth University, Richmond, VA, USA; ^5^University of Rwanda, College of Medicine, Kigali, Rwanda; ^6^Virginia Commonwealth University School of Medicine, Richmond, VA, USA; ^7^Department of Health Behavior and Policy, Virginia Commonwealth University, Richmond, VA, USA; ^8^Program for Global Surgery, Department of Surgery, Virginia Commonwealth University, VCU Health, Richmond, VA, USA

## Abstract

**Introduction:**

Pain is a universal human experience tied to an individual's health but difficult to understand. It is especially important in health emergencies. We performed a two-step quality improvement project to assess pain management by the SAMU ambulance service in Kigali, Rwanda, examining how pain is assessed and treated by ambulance staff to facilitate development of standardized guidelines of pain management in the prehospital setting, which did not exist at the time of the study.

**Materials and Methods:**

Deidentified ambulance service records from December 2012 to May 2016 were analyzed descriptively for patient demographics, emergency conditions, pain assessment, and medications given. Then, anonymized, semistructured interviews of ambulance staff were conducted until thematic saturation was achieved. Data were analyzed using a grounded theory approach.

**Results:**

SAMU managed 11,161 patients over the study period, of which 6,168 (55%) were documented as reporting pain and 5,010 (45%) received pain medications. Men had greater odds of receiving pain medications compared to women (OR = 3.8, 95% CI (3.5, 4.1), *p* < 0.01). Twenty interviews were conducted with SAMU staff. They indicated that patients communicate pain in different ways. They reported using informal ways to measure pain or a standardized granular numeric scale. The SAMU team reviewed these results and developed plans to modify practices.

**Conclusions:**

We reviewed the existing quality of pain management in the prehospital setting in Kigali, Rwanda, assessed the SAMU staff's perceptions of pain, and facilitated standardization of prehospital pain management through context-specific guidelines.

## 1. Introduction

Pain is an unpleasant sensory and emotional experience associated with actual or potential tissue damage [1]. It is an inherently subjective concept, perceived, experienced, and expressed differently by different groups of people [2–6]. Pain is often undertreated by health professionals, leading the World Health Organization (WHO) and many international organizations to advocate for treatment of pain [7]. WHO's landmark three-step ladder for cancer pain relief in adults is now widely applied to manage all types of pain in both emergent and nonemergent settings [8].

Treatment of acute pain in emergency care gets insufficient attention [9]. Pain management can be challenging because people express their pain so differently, and it can be challenging to assess [10–13]. A Dutch study identified that only 19–30% of trauma patients receive pharmacologic pain treatment [14]. Acute pain can cause adverse outcomes in patients such as alter cardiovascular function, impede normal pulmonary function, cause immobility, and contribute to increased morbidity [15]. As such, it is critically important to treat patients' pain. Despite this need, there is a lack of literature regarding pain tolerance in the majority of Africa, and pain in the acute setting has rarely been described [16]. The current study seeks to fill this gap in the literature.

An area once faced with an incredible burden of infectious disease, with improved treatments, sub-Saharan Africa (SSA), now suffers from an increasing prevalence of injury [17, 18]. Despite this burden of injury, SSA has the lowest consumption of opioid medication worldwide [19–21]. One of the reasons for inadequate pain control could be due to the lack of early interventions and prehospital care [22]. While there is some evidence that paramedics perceptions of patients' pain expression differs based on culture, few qualitative studies have examined the perception of patients' pain expression from the EMS providers' perspective and none from Africa [23].

Within SSA, Rwanda has a thriving prehospital Ministry of Health sponsored ambulance service, Service d'Aide Medicale d'Urgence (SAMU), making it an ideal case study. This study's aims are to explore the pain experiences and expressions of acutely ill patients on the ambulance in Rwanda from the perspective of those working on the ambulance, investigate emergent pain diagnosis and management on the ambulance in Rwanda, and to develop standardized pain management care guidelines for SAMU staff. This manuscript reports on the conduct of this quality improvement project and its results.

## 2. Methods

### 2.1. Study Context and Setting

SAMU provides emergency care to the public and serves as the only emergency medical service in Rwanda. SAMU is a division of the Ministry of Health of Rwanda and is fully funded by the government but requires patients to pay a small fee for their service. Ambulances are staffed by nurses and nonphysician anesthetists who have training for in-hospital care. They have received no formal prehospital training, as these programs do not exist in Rwanda. Nurses and nonphysician anesthetists deliver emergency prehospital care through formalized procedures developed as part of a collaboration between SAMU and Virginia Commonwealth University [24]. These protocols were recently developed, and the pain protocol described in this manuscript was included. Prior to their development, there were no formalized guidelines. Staff members provide care and transportation in Rwanda's capital city of Kigali and surrounding areas. Both nurses and nonphysician anesthetists are allowed to administer all available medication to patients on the ambulance. They work with a team of staff and emergency physicians to review their cases each day and improve their care. Healthcare professionals perform triage, provide stabilizing interventions, and make decisions regarding transportation. SAMU has previously been described in detail [25].

### 2.2. Quantitative Analysis

#### 2.2.1. Study Design

This is a cross-sectional study of patients managed by SAMU between 2012 and 2016 in Kigali, Rwanda. A long standing deidentified electronic registry (REDCap, Vanderbilt University; Nashville, Tennessee, USA) was reviewed retrospectively for variables of interest that were routinely captured during clinical care [25].

#### 2.2.2. Variables

Gender, age, and chief complaints were analyzed. Urgency was subjectively assigned as absolute, relative, or no urgency based on a number of factors including vital signs and patient appearance. Pain was classified as yes or no in the registry. There is no formal pain scale used by SAMU during standard clinical care. Records met criteria for inclusion in this study if they documented pain or the administration of pain medication even if pain was not explicitly documented. Other variables included were body location of pain and medications provided. We reviewed the data to evaluate when pain medication was given and under what circumstances, focusing on both patients who reported pain and patients who were provided pain medications.

### 2.3. Data Analysis

Descriptive and comparative analyses were performed using SPSS version 25. Continuous variables were compared using *t*-tests, while categorical variables were compared using chi-squared and Fisher's exact tests. An alpha of 0.05 was used to determine significance. Odds ratios and 95% confidence intervals were calculated to quantify differences in the assessment and management of pain by gender. Only patients with recorded values for a given variable were included in analysis.

### 2.4. Qualitative Analysis

#### 2.4.1. Study Participants and Sample Size

SAMU nurses, nonphysician anesthetists, and ambulance drivers were recruited through convenience sampling for this anonymized portion of the project. Inclusion criteria were voluntary participation and SAMU affiliation for a minimum of one year. This research was conducted in Kigali, Rwanda, at the SAMU headquarters located within the University Teaching Hospital of Kigali (CHUK). SAMU was started in 2007 and became a formal division of the Ministry of Health in 2017. They employ 70 staff, nurses, anesthetists, and drivers. Twelve SAMU ambulances work within the city of Kigali and 270 ambulances throughout Rwanda at provincial and district hospitals [25].

#### 2.4.2. Study Design, Setting, and Analysis

An interview guide was developed with open-ended questions with follow-up prompts to gain information about the variables of interest. Interviews were 30 minutes in length on average and were audio-recorded and transcribed by two of the authors (AR/DB). Interviews were conducted in English, with a translator, and a Rwandan medical student (TC) was present for a majority of the interviews (13) to translate to the local language of Kinyarwanda if the interviewee preferred. Quotes provided by the translator may not be actual quotes from interviewees but from the translator. All interviews were transcribed verbatim and no identifying information was collected.

The transcripts were analyzed using a comparative qualitative content analysis [26]. Two authors (AR and JG) hand coded each interview transcript. During initial coding, through a detailed reading of the text, each piece of data was coded line by line, and these pieces were then labeled with codes based on beliefs, feelings, perceptions, and attitudes. Codes were identified and subsequently categorized under distinct headings. Following this process, code mapping was used to organize the codes into broader categories, linking codes to one another, leading to the identification of predominant themes. Codes that only occurred once and that did not show any consistency with more often occurring codes or categories were excluded in order to prevent information overload. The coded data were then compared to ensure that themes were consistent between both researchers, and the coding process continued until saturation was reached [27].

#### 2.4.3. Ethical Approval and Consent

This project was conducted within a Memorandum of Understanding between the Ministry of Health of Rwanda and Virginia Commonwealth University to facilitate trauma and emergency systems in Rwanda. Research ethics approvals were obtained at Virginia Commonwealth University and the University Teaching Hospital, Kigali, for analysis of the deidentified electronic prehospital registry. An additional IRB amendment was sought for incorporation of results of the anonymized interviews in this manuscript.

## 3. Results

### 3.1. Quantitative Results

#### 3.1.1. Documentation of Pain

From December 2012 through June 2016, SAMU managed 11,161 patients, of which 55% (*n* = 6,168) reported pain. The average patient reporting pain was 31.5 years old (+13), which was significantly younger than SAMU patients who did not report pain (33.5 y + 20.5) (*p* < 0.01). The majority of patients reporting pain was men (*n* = 4,661, 76%) and had 5 times the odds of reporting pain compared to women (95% CI (4.6, 5.4), *p* < 0.01).

The most common location of pain was described as the lower limb (*n* = 2,414, 39%) followed by the skull (*n* = 2,186, 35.5%). Of 6,168 patients who had pain documented, the most common cause was trauma (*n* = 5,724, 93%) followed by medical-related problems (*n* = 439, 7%). Patients reporting obstetric-related complaints were rarely documented for pain—only 8 of those reporting pain had obstetric complaints (<1%) (Table 1). During this time, there were 1,781 obstetric patients managed by SAMU.

Acuity of illness is assessed by urgency categories. Patients were triaged by SAMU into 3 urgency categories (absolute, relative, and no urgency) based on clinical presentation during field assessment. Patients who were categorized as absolute urgency had lower odds of having documented pain compared to all others (OR = 0.38, 95% CI (0.34, 0.42), *p* < 0.01). The majority of patients reporting pain (*n* = 2,468, 44%) was transported to the University Teaching Hospital of Kigali (CHUK), which is the major trauma center in the country (Table 1).

#### 3.1.2. Management of Pain

Of those who had documented pain, 4,449 (72%) patients received pain medications. An additional 561 patients who did not have pain documented also received medications, for a total of 5,010 (45%) patients overall. The average age of a patient receiving pain medications was lower (31 + 12 vs. 33.5 + 20, *p* < 0.01). The majority of patients receiving pain medications was men (*n* = 3795, 76.5%). When considering all patients managed by SAMU, men had higher odds of receiving pain medications compared to women (OR 3.8, 95% CI (3.5, 4.1), *p* < 0.01). Similarly, men who reported pain had greater odds of receiving medications compared to women who reported pain (OR = 1.5, 95% CI (1.3, 1.7), *p* < 0.01).

The most common medications were diclofenac (*n* = 3,248, 65%), morphine (*n* = 1,011, 20%), and paracetamol (*n* = 383, 7.5%). The most common location of pain was described as the lower limb (*n* = 1,951, 39%) followed by the skull (*n* = 1,695, 34%). Trauma was the leading cause of pain requiring treatment (*n* = 4,618, 92%), followed by medical-related problems (*n* = 306, 6%) and obstetric patients (*n* = 69, 1%) (Table 1). Trauma patients had greater odds of receiving pain medications compared to obstetric patients (OR = 70, 95% CI (54, 89), *p* < 0.01). Patients who were categorized as absolute urgency had lower odds of receiving pain medications compared to all others (OR = 0.52, 95% CI (0.47, 0.58), *p* < 0.01). The majority of patients that received pain medications (*n* = 2,199, 47%) was transported to CHUK.

### 3.2. Qualitative Results

Between January 16 and January 31, 2019 in Kigali, Rwanda, 20 semistructured interviews were conducted in person, which was sufficient to reach saturation as a part of a quality improvement project to understand practitioner factors in care delivery. There were 16 females interviewed, with an average age of 39 years (standard deviation (SD): 6 years). The majority of those interviewed were nurses (*n* = 12), followed by anesthetists (*n* = 7) and ambulance drivers (*n* = 1). The maximum number of years of those interviewed working in their position was 12 years, with an average of 9 years (SD: 2.5 years).

#### 3.2.1. Theme Development

In total, three main categories were identified throughout the analysis including differences in pain expression and actions taken on the ambulance. Major themes and contributions to the theme are listed in Table 2.

The first theme of pain expression can be divided into themes by age, gender, country/culture, and urban/countryside. A recurring narrative among the majority of interviewees was that older people express pain less frequently and less overtly than younger persons. Younger people are perceived to have experienced less pain in their lives, which is mentioned as an explanation for this observation. Several of the interviewees said that women express pain in a more exaggerated manner compared to men, in part due to cultural values. However, other interviewees mentioned the opposite dynamic: men expressing pain more openly than women. The most common cultural/country comparison in the interviews was between the Democratic Republic of the Congo (DRC) and Rwanda. Comparing the two cultures and how they tend to express pain, Congolese are described as expressing pain loudly and in an exaggerated manner, while Rwandans are described as not expressing pain and being patient, sometimes referring to the genocide against the Tutsi in Rwanda as an underlying reason. Urban/city residents were generally viewed as exaggerating their pain and expressing it more overtly compared to countryside residents.

The second theme, actions of the ambulance, can be divided into assessing, expressing, and managing pain. When asked how a patient's pain generally is assessed on the ambulance, most frequently, pain scores (1–10) are mentioned. In addition, interviewees mentioned patient observation as a pain assessment technique and vital signs such as blood pressure and heart rate. Relating to pain management on the ambulance, most interviewees refer to administering different types of pain medication: paracetamol/acetaminophen (i.e., Tylenol) or ibuprofen, diclofenac, or morphine. The interviewees mentioned a number of different pain expressions by patients—most frequently crying, screaming, and facial expressions. Most of the healthcare professionals interviewed for this study mention that the pain medication they need is always available. However, they do mention some situations where administering pain medication is problematic: among them, patients refusing pain medication because of fear of needles and patients with abnormal vital signs.

#### 3.2.2. SAMU Team Review

**Figure 1:**
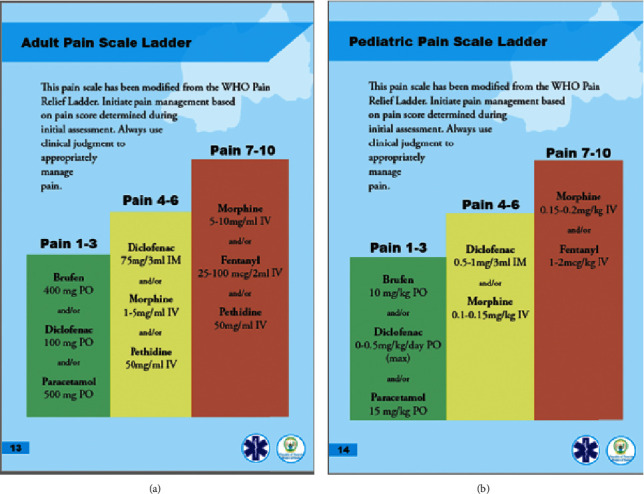
Adult and pediatric pain protocols.

Results of both assessments of pain management in the prehospital setting and staff attitudes and perceptions of pain were reported back to the entire team by one SAMU staff member for discussion. Novel pain management guidelines for the service were drafted collaboratively based on the WHO's standardized pain scale [8, 28]. This was shared with each staff member as both a pediatric and adult pain protocol section of a larger document to standardize care on the ambulance (Figure 1). Additionally, ambulance run sheets were updated to include more detailed information about pain scores and management in the field during all future patient encounters.

## 4. Discussion

This study is one of the first to explore pain expression or provide epidemiologic characteristics relating to pain management in the prehospital setting in SSA [16]. While research indicated that pain is widely experienced in the emergency setting, pain is expressed nonuniformally, which affects its treatment. The majority of those recording pain in this analysis were injured, followed by those having medical emergencies with less than 1% being related to obstetric causes. Significantly more men reported pain compared to women, and men were more likely to receive pain medication than women. In the interviews, SAMU staff reported culture and gender as important factors affecting the expression of pain, with Congolese perceived to be exaggerating their pain compared to Rwandese and women compared to men. Additionally, they reported that rural patients were less likely to express pain than city dwellers.

This study demonstrates differences in the pain documentation, treatment, and expression for males and females. The quantitative data showed that the majority of patients documented as having pain was men. Men were also more likely to receive medications compared to women. This was contradictory to the qualitative analysis where women were perceived as exaggerating pain compared to men. This may suggest some implicit bias. SAMU staff view pain as being expressed differently in men and women, which is supported throughout the literature. For example, studies have found different pain modalities to be perceived differently by men and women [28, 29]. Although some variations in pain expression exist, gender-based treatment of pain may have a negative impact on patient care such as fewer referrals to specialist physicians [29, 30]. Studies specific to analgesics administration in the prehospital setting have shown that women often receive less medications than men for similar injuries [31, 32]. Our findings support previous work that suggests a bias in provider treatment of pain based on sex. This may lead to under treatment of female patients. Providers must be aware of these potential biases and the impact it can have on patient care in order to improve pain management.

Another major difference in the assessment and management of pain was the cause of pain. The majority of patients documented as having pain was from injury, although clearly other conditions such as labor can be painful. The number of patients with obstetrics-related complaints documented as having pain was very low. Our qualitative interviews did not ask which chief complaints would require pain medication, so it is unclear if SAMU staff would normally ask every patient if they are in pain when responding to emergencies. However, undertreating labor pain has been raised as a common issue in similar settings. A study from Ethiopia reported that pain medications were used for labor and delivery in only 34% of patients [33]. In Nigeria, a study examining pain in women in labor found that they reported having severe pain and would be interested in pain relief during labor if available [34]. Understanding that differences in pain management based on the cause of pain exist, provides additional data for the SAMU team to improve prehospital care.

Cultural background clearly can influence how people perceive, report, and manage pain. Some may permit the expression of pain while others forbid such a public expression or express it in different ways [35]. These differences were captured in the qualitative analyses. In particular, the SAMU staff discussed differences in the way pain was expressed between those from the DRC compared to patients from Rwanda. Patients from the DRC were described as more likely to exaggerate their pain and express it through shouting or crying than Rwandese. Different cultures have been found to express pain differently with some forbidding its expression [35]. This is consistent with the differences expressed by SAMU staff regarding Rwandan and Congolese patients and makes prehospital pain management complicated in Rwanda.

Last, since pain is a subjective experience, the SAMU team's attitudes towards pain also affect assessment and management of pain in the prehospital setting. Historically, SAMU has had no established way of diagnosing and assessing pain. This has led to heterogenous practices among the staff. For example, some of those interviewed at SAMU were unaware of the pain scale and were not familiar with its use in choosing a pain medication for treatment. Although limited research on prehospital pain management has been performed in sub-Saharan Africa, studies have shown limited pain assessment or use of pain scores [16]. Using a well understood pain scale, however, can help improve the assessment of pain and combat cultural differences [36, 37]. The WHO recommends the use of the pain scale ladder for more uniform assessment and treatment of pain [8, 28]. This study has led SAMU to incorporate the WHO pain scale into their standard patient assessment. This includes specific information regarding which medications should be given at each pain level to help mitigate differences in pain assessment and treatment and standardize overall management.

This study has a number of limitations. First, the quantitative data regarding documentation and management of pain were from EMS records. These data were not collected specifically for this study. Data may have been missing or incompletely documented. Data are limited to prehospital care and is not linked to emergency department or in-hospital data. We have no outcomes data to see the patient's formal diagnosis or hospital course. The study was an analysis of the current pain treatments but not as much adequacy of care, which will be further studied in the future. Additionally, the qualitative interviews were conducted by a nonnative interviewer which may have created language and cultural barriers, which may have biased results. A Rwandan translator (TC) was assisted with the interviews; however, barriers may have remained. Furthermore, having a male translator may have led to bias on how the staff considered gender in relation to pain and how they spoke to him about it. Furthermore, interview-based data collection can be subject to various sources of participant and researcher bias. Since the interviews were conducted by members who were well known to the SAMU staff and had long-term relationships with participants, staff may have been more open in their responses. However, in this case, acquiescence and social desirability bias on behalf of the SAMU staff and confirmation bias on the part of the interviewing team could have biased the results in various ways. The optimal method to address this would be to conduct follow-up studies including interviewing patients, independent of the treating staff, to get their views on pain expression.

## 5. Conclusion

Pain is a personal experience; it is a difficult phenomenon to express and treat. This was the first study to examine pain expression in the prehospital setting in Rwanda and one of the only studies to do so in Africa. This study opened a conversation at SAMU about why the treatment of pain is important and how pain can be expressed uniquely in different populations. The results were used to develop context-specific interventions to standardize prehospital pain management as part of a quality improvement effort at SAMU.

## Figures and Tables

**Table 1:** Pain etiology and location by patients with documented pain and those who received pain medications.

Pain characteristic	Patients with documented pain (%)	Patients who received pain medications (%)
Total	(*n* = 6,168)	(*n* = 5,010)
Age	31.5 y (±13)	31 y (±12)
Sex		
Male	4,661 (76)	3,795 (76.5)
Female	1,457 (24)	1,172 (23.5)
Etiology		
Trauma (total patients = 6,266)	5,724 (93)	4,618 (92)
Road traffic incidents	4,308 (70)	3,578 (71.5)
Assaults	652 (10.5)	485 (9.5)
Fall	452 (7.5)	330 (6.5)
Burns	60 (1)	53 (1.0)
Others	249 (4)	112 (2)
Medical (total patients = 3,062)	439 (7)	306 (6)
OB-GYN (total patients = 1,781)	8 (0.1)	69 (1.5)
Location of pain		
Lower limb	2,414 (39)	1951 (39)
Skull	2,186 (35.5)	1304 (26)
Face	1,701 (27.5)	1695 (34)
Upper limb	1,380 (22)	1069 (21.5)
Chest	861 (14)	551 (11)
Spine/back	477 (7.5)	372 (7.5)
Others	609 (10)	367 (7)
Acuity		
Absolute	637 (10.5)	567 (11.5)
Relative	4,431 (73.0)	3,713 (75.5)
No urgency	998 (16.5)	646 (13.0)

**Table 2:** Qualitative themes, families.

Major theme	Code families included	Sample codes included
Differences in pain expression	Age	“Mostly young people cry and shout when there is any pain. But others try to manage it and be patient. Young people are immature, and once they find blood from anyone, they get upset and cry.” The way the youth express pain nowadays is different from the others; they express in an exaggerated way. The reason is because the youth have not had many experiences of pain. There is the example of genocide for the older people. So, when they have pain, they compare the pain they are feeling at that time with the pain they had during genocide, and then, they try to calm themselves. But for the youth (unclear: to have the understanding?) is by sharing the story of how genocide happened because they did not pass through that kind of life.”
	Gender	“For the females, they obviously try to express what they have in their heart, so you know they are in pain. Also, in the Rwandan culture, the tears of men move down to inside and are not coming outside. Which causes them when they are in their environment, they do not express they are crying because then people will think he is not a manly man because he is crying.” “Men do not express pain the way females do; females exaggerate it. Rwandan culture says men are not supposed to express pain by crying and shouting like how females do. The culture makes men resist.” “Mostly the men express their pain more than women. Women are exposed to pain many times during labor, and they compare their pain, but men do not.” “Men express pain more obviously, and everyone can know the man is in pain.”
	Country/culture	“Rwandans express pain differently from Congolese. The Congolese even in labor, they shout and make people know they are in pain. But the Rwandese, most people especially those who are older, they try to make things seem calm. They try to calm themselves and say that there is no other way.” “Congolese like to cry. They like to express their pain even if the pain is moderate or mild. We say they exaggerate to express their pain. As I told you before, in Rwandese, you can find someone with femur fracture who is not crying, without expressing pain, and being calm, until you touch or move the limb that is fractured.” “I think it is because of the culture of Rwandese; they like to hide their emotion compared to Congolese.”
	Urban/countryside	“In the countryside, the reason why they do not express their pain is because they fear people from the city and so do not show pain. Also, their character. The people from countryside are closed compared to city people. In the city, you can talk more, and you can say anything. But the people from countryside are always shy. They need to have much time to assess people from countryside because sometimes they say they do not have pain when they actually are having pain.” “… for those in the countryside, they meet with many things in life which hurt them, which cause pain for them. So, it is like to have a pain but it is not pain compared to those in the city who have the easiest life to enjoy always, not having many hard things.”

Actions on the ambulance	Assess pain	“They use pain scores. Sometimes they use inspection. If the patient has chest pain and shouting, then they know that maybe this patient has 8 out of 10 pain. And also, they look at vital signs: blood pressure is going high and heart rate is abnormal. So, the patient uses signs to show they are in pain.” “First, they use inspection, looking at how the patients look, mostly the face. Those who have weak or shrunken face have severe pain. Then, second, after inspection, they use palpation. When they palpate painful site, the patient tells them not to palpate on that site.”
	Manage pain	“For minor injury, give paracetamol and ibuprofen. For those who have moderate injury, you give diclofenac but not if bleeding. If severe pain, they give morphine in a titrated way: they give 4 mg and then assess.” “We decide to give painkiller depending on many things. First of all, we look for the scale; even if I do not use the scale, I can evaluate the pain based on the expression. We can also look at vital signs. For example, traumatic patient in shock. If he has pain and is crying, the vital signs will show that the patient is in shock. To decide which painkiller, I have to look at vitals because some painkillers reduce cardiac output.”
	Patient expression of pain	“They express in three ways: patients who talk when in pain, others show on face that they are in pain, and then there are those who shout from pain.” “It depends on personality and severity of pain. Some people have the personality that once they have pain, they exaggerate it. There are others who make it simple even though they have severe pain. Also, there are some with such severe pain that they are not able to resist the pain and so they shout and cry out.”
	Ability to provide medication	“Some refuse injection, they do not even want to see the needle. They try to counsel the patient. If they keep refusing, they do not give. Others might want water to swallow the medication but there is no water in the ambulance. So, some do not want an injection, but even for the tablet, they need water to swallow.” “Because they fear medication and refuse it. Sometimes, the patient refuses to go to the hospital or care facilities. They think the medication will be poisoned by their neighbors.”

## Data Availability

The data used to support this study are made available from the corresponding author upon request.

## Appendix

Interview guide questions.

  Question 1: although everyone feels pain, people express their pain in different ways. How do SAMU patients express their pain, if at all?
  Question 2: what affects how your patients express their pain?
  Question 3: how do patients communicate about their pain to you?
  Question 4: what are some ways you can tell when a patient is in pain?
  Question 5: how often do you assess a patient's pain?
  Question 6: are there types of patients where it is harder to assess their pain?
  Question 7: can you please walk me through the way you assess a patients' pain?
  Question 8: can you list for me all the ways you can address patients' pain as a nurse/nurse-anesthetist on a SAMU ambulance?
  Question 9: you mentioned that one option is to give pain medications. How do you decide to give them?
  Question 10: is there ever a time when you want to give pain medication and you cannot?
  Question 11: do you talk to patients about giving pain medication?
